# Bronchial asthma in developing countries: A major social and economic burden

**DOI:** 10.4103/1817-1737.39633

**Published:** 2008

**Authors:** Mohamed S. Al-Hajjaj

**Affiliations:** *Respiratory Division, Medical Department, College of Medicine, King Saud University, Riyadh, KSA*

Bronchial asthma is a disease that is becoming a major health issue in many developing countries. Many factors may have contributed to the rise of the problem of bronchial asthma. Increasing air pollution, fast modernization, and widespread construction work are some of the reasons for asthma to thrive. The situation is complicated by poor access to medical services, high price of effective drugs, and poor health education among the affected population. Increased urbanization may have modified the traditionally low incidence of bronchial asthma in the Third World. Diets becoming more westernized, improvement in standard of living, decrease in exercise rates, more dust mites, and more pollution have been blamed.[1]

There have not been reliable epidemiological studies to define the magnitude of the problem in many developing countries. Rates vary between 3% and 30%, depending on the location and methods of survey. The authors of the International Study of Asthma and Allergies in Childhood (ISAAC) have reported their results of phase III of the worldwide study on the trends in the prevalence of asthma symptoms. Findings indicate that international differences in the prevalence of symptoms of asthma have decreased, particularly in the age group of 13 to 14 years. They concluded that increases in the prevalence of symptoms of asthma in Africa, Latin America, and parts of Asia indicate that the global burden of asthma is continuing to rise, but the global differences in prevalence are decreasing.[2]

In drug therapy, there are often major misunderstandings and lack of awareness among asthma patients about bronchial asthma. Many patients have steroid phobia. Others have fear of side effects or getting ‘used to’ or ‘addicted to’ inhalers, and many others may overuse or abuse these medications.

Updated guidelines on bronchial asthma have been recently issued by two major international bodies, namely, GINA and NAEEP.[3,4] Both have not addressed the problems related to developing countries. It is therefore of utmost importance that medical societies and health authorities adopt a local version of such guidelines to stress upon aspects of the disease that are related to local practices or to situations specific to local population. Use of herbal remedies or local nonconventional practices that may not be effective or may be even harmful to patients should be discouraged. Other issues are the need to address the availability and the prices of new asthma drugs and to try to choose less expensive forms or sources of medications.

Watson *et al.* investigated the applicability of therapeutic aspects of published international asthma management guidelines in developing countries. They concluded that many asthma patients in developing countries are not receiving adequate treatment because the required drugs are not available in their area or are prohibitively expensive.[5]

Therefore, major responsibility lies upon the shoulders of health authorities and local medical societies to set up continuous health education programs to improve asthma management and to clarify to patients the merits of the drugs used for treatment and to alleviate any fears of using them.

The Saudi Thoracic Society has established continuous and sustained programs focusing on educating the general practice doctors with regard to the most appropriate and recommended management of bronchial asthma. Local guidelines are currently under preparation and will be published in the coming few months. Public education is being addressed by printing and distribution of thousands of simple and easy-to-understand brochures.
